# Endothelial-smooth muscle microgauges for modeling pulmonary arterial vasoregulation

**DOI:** 10.1039/d5lc00474h

**Published:** 2025-10-13

**Authors:** Aanya Sawhney, Raymond Piatt, Mitesh Rathod, Ryan N. Stack, Chloe P. Whitworth, William J. Polacheck

**Affiliations:** a Lampe Joint Department of Biomedical Engineering, University of North Carolina at Chapel Hill and North Carolina State University Chapel Hill and Raleigh North Carolina USA polacheck@unc.edu; b Department of Genetics and Molecular Biology, University of North Carolina at Chapel Hill School of Medicine Chapel Hill North Carolina USA; c Department of Cell Biology and Physiology, University of North Carolina at Chapel Hill School of Medicine Chapel Hill North Carolina USA; d McAllister Heart Institute, University of North Carolina at Chapel Hill School of Medicine Chapel Hill North Carolina USA

## Abstract

Pulmonary arterial hypertension (PAH) is a devastating disease for which there is no cure. The pathogenesis of PAH involves endothelial dysfunction and dysregulation of vascular tone, resulting in progressively narrowing pulmonary arteries that increase hemodynamic resistance and blood pressure. The development of effective therapeutics for PAH is hindered by limitations to animal models and a lack of humanized *in vitro* systems that recapitulate endothelial-dependent regulation of smooth muscle cell contractility. Here, we microfabricated pulmonary artery smooth muscle microgauges (PA-SMUGs) that enable quantification of contractile forces generated by human pulmonary arterial smooth muscle cells (PASMCs) within microtissues that contain a functional monolayer of pulmonary arterial endothelial cells (PAECs). PA-SMUGs demonstrate PAEC-dependent vasorelaxation and respond to treprostinil, a clinically approved PAH therapy. This platform, which establishes a high-throughput method for quantifying EC-dependent vasorelaxation, will facilitate mechanistic studies into the role of PAEC-PASMC crosstalk in PAH pathogenesis and enable screening for novel therapeutics to improve PAH outcomes and hypertensive diseases more broadly.

## Abstract 2

A pulmonary artery-on-chip platform self-assembles, responds to hemodynamic flow, and provides quantitative measurements of endothelial-dependent vasoconstriction and dilation in control and pulmonary arterial hypertension patient donor cells.

## Graphical abstract

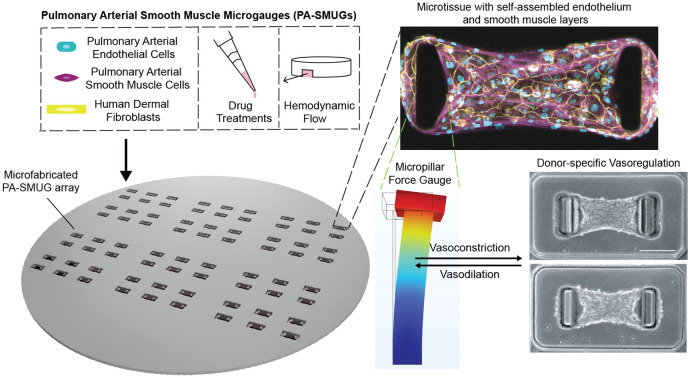

## 1. Introduction

Vascular smooth muscle cell (VSMC) contractile activity modulates vascular tone of arteries and arterioles to determine peripheral vascular resistance, regulating blood pressure and flow.^1^ Dysregulation of VSMC contractility along with increased proliferation and extracellular matrix (ECM) deposition contribute to several disease states, including pulmonary arterial hypertension (PAH).^2,3^ In arteries, VSMCs reside in the tunica media of the vessel wall and are surrounded by a collagen- and proteoglycan-rich ECM.^4^ VSMCs are principally responsible for the generation of the mechanical forces necessary to modulate vessel diameter. However, dynamic and reciprocal biophysical and biochemical interactions between VSMCs and the cells and ECM of the intimal and adventitial layers of the arterial wall collectively regulate vasoconstriction and dilation.^3,5,6^ Thus, dissecting cellular contributions to pathologies in which arterial tone is dysregulated is difficult *in vivo* due to the interdependencies of these layers, and engineered and reductionist approaches have improved understanding of the genetic and molecular regulators of vascular tone in health and disease.^7^

PAH is a severe lung condition in which elevated pulmonary arterial pressure leads to hypertrophy of the right ventricle, which can eventually lead to right ventricular failure and death if untreated.^8,9^ Endothelial injury and dysfunction are hallmarks of PAH,^10,11^ contributing to an imbalance in the regulation of vascular tone that favors increased vasoconstriction and drives an increase in pulmonary vascular resistance.^12,13^ Current therapeutic approaches seek to correct endothelial dysfunction and restore homeostatic endothelial-VSMC crosstalk and vasoregulation.^8,14^ While several therapies targeting the endothelin 1, prostacyclin (PGI_2_), and nitric oxide pathways have been developed,^8^ these therapies largely mediate symptoms *via* pulmonary vasodilation,^15^ and there remains no cure.^16^ A challenge in the development of novel therapies is that animal models do not fully recapitulate the disease.^17^ To address this challenge, humanized *in vitro* approaches have been developed to investigate endothelial cell (EC-) VSMC crosstalk, broadly categorized into three strategies:^7^ 2D culture of ECs directly on top of VSMCs,^18–20^ co-culture of EC and VSMC monolayers on either side of a permeable membrane,^21–23^ and culture of ECs on 3D hydrogels embedded with VSMCs.^24,25^ While these approaches have been critical in identifying molecular mediators of EC–VSMC signaling and crosstalk, assessment of VSMC contractility has largely been inferred indirectly *via* biochemical analyses of gene and/or protein expression, which presents a challenge toward understanding and treating the mechanics of vasoconstriction and dilation in PAH.

Several techniques have been developed to measure cell-generated forces that govern regulation of vascular tone in resected arterial tissue and isolated VSMCs.^26^ Myography is the gold standard technique for quantifying pharmacological mediation of vasoconstriction and dilation,^27^ yet the requirement for resected arterial or venous tissue from animal models limits applicability for PAH. Traction force microscopy (TFM) and micropillar arrays allow quantification of cell-generated forces by cultured human cells on engineered substrates,^28^ and have been used to measure contraction of VSMCs,^29–33^ yet these cell-based assays lack EC co-culture. While microfabricated tissue-scale assays have sought to bridge the gap between cell-based assays and resected tissue through the determination of cell-generated forces as a surrogate for SMC-mediated vasodilation,^34,35^ these approaches lack a functional endothelium^35^ or were constructed in a manner that does not allow quantification of vasodilation.^34^

To address shortcomings with these previous approaches, here we adapt microfabricated microtissue gauges (TUGs),^36^ which have been used to quantitatively determine contractile force dynamics of fibroblasts,^37^ skeletal muscle,^38,39^ cardiac muscle,^40^ and airway smooth muscle,^35^ among other tissues.^28^ We fabricated master molds using photolithography and monolithically replica molded gauges using soft lithography, resulting in devices that provide high-throughput determination of contractile forces generated by pulmonary arterial smooth muscle cells (PASMCs), including PASMCs from human PAH donors. The resulting smooth muscle microgauges (SMUGs) allow quantification of PASMC-generated forces in 3D microtissues, and co-culture with pulmonary arterial endothelial cells (PAECs) results in microtissues with distinct endothelial and smooth muscle compartments, reminiscent of the arterial wall. Importantly, the EC layer presents a functional diffusive barrier, and SMUGs demonstrate EC-dependent vasorelaxation. Thus, the approach and devices described here provide a novel platform for further investigation of the cellular and molecular mechanisms that underlie PAH and for screening interventions to ameliorate pathologic vasoconstriction driving elevated arterial pressure and associated morbidity and mortality of PAH.

## 2. Results

### 2.1 Microfabricated tension gauges enable measurement of cell-generated forces

To recapitulate smooth muscle cell vasoregulation *in vitro*, we sought to quantitatively measure cell-generated forces from microtissues comprised of VSMCs in collagen-rich 3D ECM. Previously, TUGs with cantilevers characterized by 0.098 and 0.397 μN μm^−1^ stiffness values were used to measure contractility of tissues comprised of airway smooth muscle cells (ASMCs) and 3 T3 fibroblasts.^35^ To develop cantilevers with the appropriate stiffness for resolving PASMC-generated forces while allowing for EC–VSMC crosstalk, we determined the expression of calponin, prostaglandin I2 receptor (PTGIR), and platelet endothelial cell adhesion receptor 1 (PECAM-1) by PASMCs, human bronchial smooth muscle cells (BSMCs), human dermal fibroblasts (HDFs), and PAECs. All cell types expressed PTGIR, while PECAM-1 was restricted to PAECs, and calponin was expressed by all non-EC cell types (Fig. S1). To determine baseline contractility and functional effects of PTGIR activation, we conducted a collagen contraction assay in non-adherent well plates and microfluidic devices with BSMCs and PASMCs embedded in 2.5 mg mL^−1^ collagen type I hydrogels. We found that both cell types contracted the hydrogels by similar magnitudes and that contraction was attenuated by treatment with the PTGIR agonist treprostinil (Fig. S1). These bulk assays reported a higher magnitude and rate of contraction compared to previous reports of ASMC contraction in hydrogels.^41^ Thus, we expected higher magnitudes of cell-generated forces and contraction by PASMCs compared to ASMCs, so we fabricated SMUGs with stiffer cantilevers ranging from 0.52–1.45 μN μm^−1^ (Fig. S2 and S3).

**Fig. 1:**
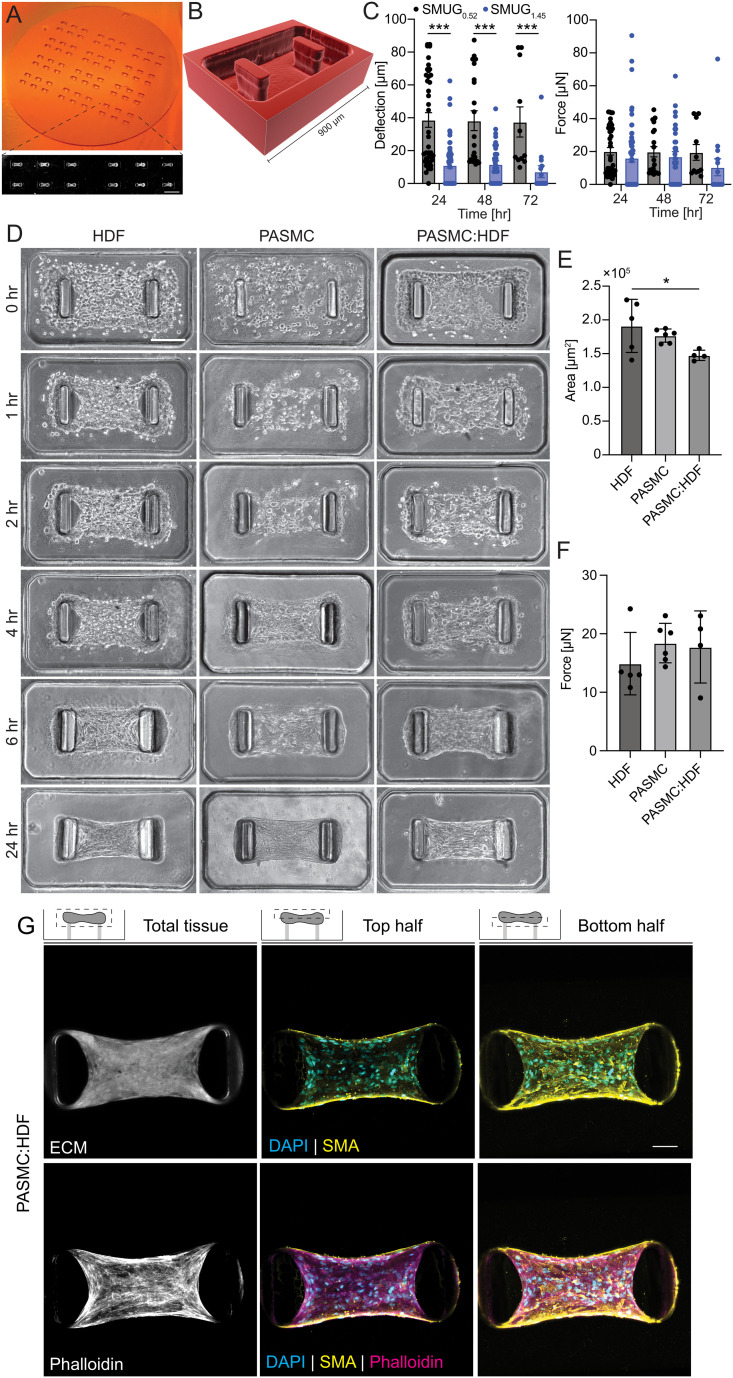
Characterization of pulmonary arterial smooth muscle microtissue formation, contractile force, area, and organization. (A) Image of SMUG_0.52_ microfabricated silicon master mold (diameter of stamp is 25 mm) with inset micrograph of 2 × 3 array of microtissues seeded with HDFs after contraction (scale bar 0.9 mm). (B) 3D reconstruction of confocal images of Nile red-labeled PDMS microwell prior to cell seeding. (C) Post deflection and forces computed with post bending stiffness for microtissues seeded with 5 × 10^5^ HDFs mL^−1^. (D) Representative phase-contrast images of microtissue formation time course of HDF, PASMC, and PASMC : HDF (4 : 1). All conditions seeded at 5 × 10^5^ cells per mL (scale bar 0.24 mm). Quantification of (E) projected area of microtissues 24 h after seeding and (F) contractile forces generated by microtissues. (G) Confocal maximum intensity projections of PASMC : HDF (4 : 1) microtissues projected for the whole tissue, top half, and bottom half as indicated by schematic (scale bar 100 μm). All plots are mean ± S.E.M. with each datapoint representing an individual microtissue, statistics determined by one-way ANOVA, *n* ≥ 4 microtissues, *p < 0.05, ***p < 0.001.

We used multilayer microfabrication to generate SMUG silicon master molds consisting of arrays of 72 0.9 × 0.5 mm wells containing rectangular cantilevers separated by 0.5 mm (Fig. 1A). To adjust cantilever stiffness without changing well volume, we fabricated SMUGs with cantilevers of varying geometry, and we designed the tops of the cantilevers to flare outward to minimize microtissue detachment during cantilever deflection (Fig. 1B). To ensure a dynamic range that allows for determination of cell-generated forces by cantilever deflection, we seeded SMUGs with HDFs in collagen type I hydrogels, and we observed significantly different deflection magnitudes in SMUGs with a bending stiffness of 0.52 μN μm^−1^ (SMUG_0.52_) *vs.* 1.45 μN μm^−1^ (SMUG_1.45_), though the calculated force was not significantly different between the two devices (Fig. 1C). Furthermore, we observed that the cell-generated forces remained constant from 24 to 72 h, though after 72 h, we observed attrition of microtissues on more compliant cantilevers due to cantilever deflection (Fig. 1C).

### 2.2 PASMC form contractile microtissues

Previous work has demonstrated that inclusion of fibroblasts is necessary for stable tissue assembly with ASMCs, and without fibroblasts, ASMCs form tissues with gaps at the tissue-cantilever interface.^41^ Informed by this work, we seeded SMUGs with PASMCs with and without fibroblasts at a cell ratio of 4 : 1 PASMC : HDF as previously described^41^ (Fig. 1D). While we did not observe gaps at the tissue-cantilever interface without fibroblasts, we found that the inclusion of fibroblasts resulted in more compact microtissues as measured by projected area (Fig. 1E). By measuring cantilever deflection and computing the magnitude of cell-generated forces, we observed that PASMCs generated similar magnitudes of force as compared to HDFs and that PASMC : HDF co-culture did not significantly impact the magnitude of forces generated at 24 h (Fig. 1F). To determine the distribution of PASMCs in compacted microtissues, we labeled the ECM by conjugating free lysines with AlexaFluor 647 (ref. 42) and stained microtissues with phalloidin for filamentous (F-) actin, α-smooth muscle actin (αSMA), and 4′6-diamidino-2-phenylindole (DAPI). While phalloidin-positive cells were found throughout the microtissue, αSMA localized to the bottom surface of the microtissues (Fig. 1G), suggesting that PASMCs form a contractile layer at the bottom surface of the microtissues.

### 2.3 SMCs derived from patients with PAH exhibit baseline contractility similar to healthy controls

**Fig. 2:**
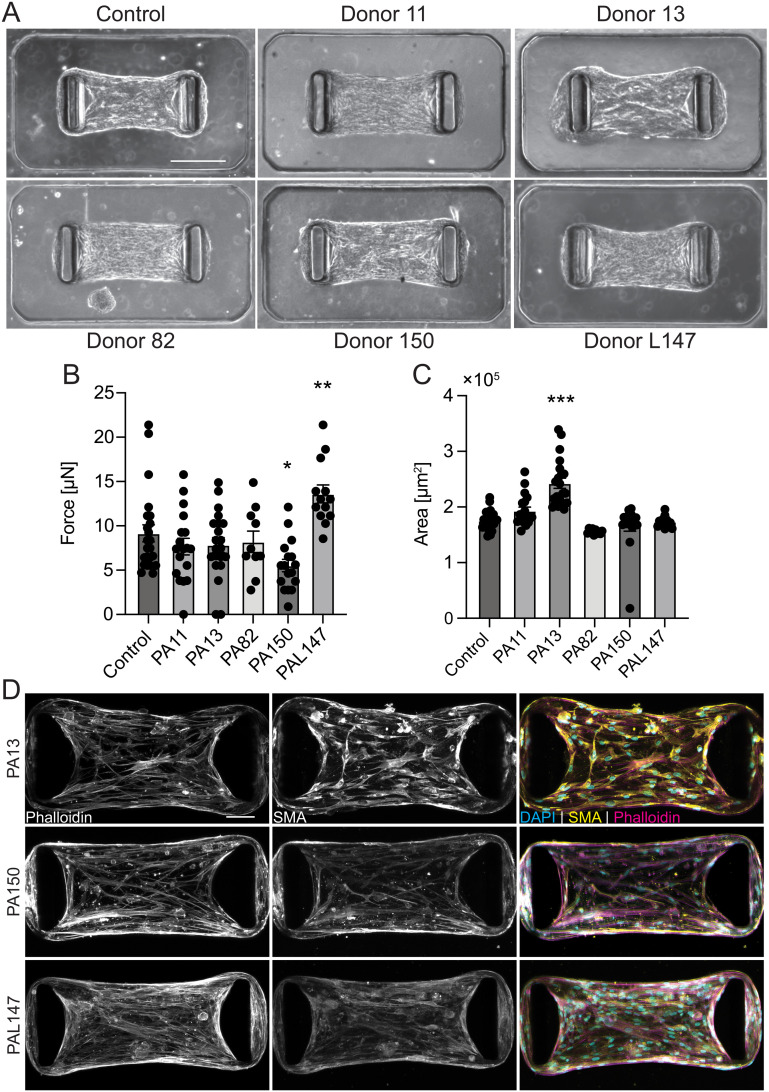
Characterization of donor-derived pulmonary arterial smooth muscle microtissue force and area. (A) Representative phase-contrast images of 24 h endpoint control and donor PASMC : HDF (4 : 1) microtissues, seeded at 5 × 10^5^ cells per mL slurry (scale bar 0.24 mm). (B) Quantification of control and donor PASMC : HDF (4 : 1) microtissue force and (C) projected area 24 h after seeding. (D) Representative images of microtissues from donors with forces or areas that differ significantly from baseline 24 h after seeding (scale bar 75 μm). All plots mean ± S.E.M., *p < 0.05, **p < 0.01, ***p < 0.001 *vs.* control as determined by one-way ANOVA, *n* = 10 microtissues.

We next sought to determine whether PASMCs obtained from donors with clinically confirmed PAH demonstrated differential baseline contractility as compared to healthy control PASMCs. We acquired PASMCs from 5 donors with confirmed idiopathic pulmonary arterial hypertension (IPAH) from the Pulmonary Hypertension Breakthrough Initiative (PHBI, Table S2, SI Methods). While there was heterogeneity among microtissues formed from individual donors, there was no consistent difference in baseline contractility or microtissue area in the IPAH donor PASMCs compared to the control (Fig. 2). Interestingly, staining for DAPI, phalloidin, and αSMA revealed differences in cytoskeletal structure in microtissues that demonstrated areas and forces significantly different from control. Phalloidin staining in microtissues from donor 150, which produced less force than the control, showed larger voids between F-actin filaments in microtissues. (Fig. 2D). Conversely, microtissues from donor L147, which produced more force than the control, featured tightly distributed F-actin fibers (Fig. 2D). Voids were also seen between F-actin fibers in microtissues from donor 13. However, these microtissues produced similar forces to the control, despite having a loose and malformed microtissue structure as observed by phase contrast (Fig. 2A–D) Additionally, staining for αSMA in microtissues from donor 13 revealed a more intense signal than other donor samples (Fig. 2D). The lack of a consistent deviation in force or area of donor microtissues as compared to control, and clinical data demonstrating the critical role of ECs in PAH progression suggests that functional ECs are required to recapitulate aberrant vasomodulation in PAH.

### 2.4 PAEC form a functional monolayer at the surface of microtissues

**Fig. 3:**
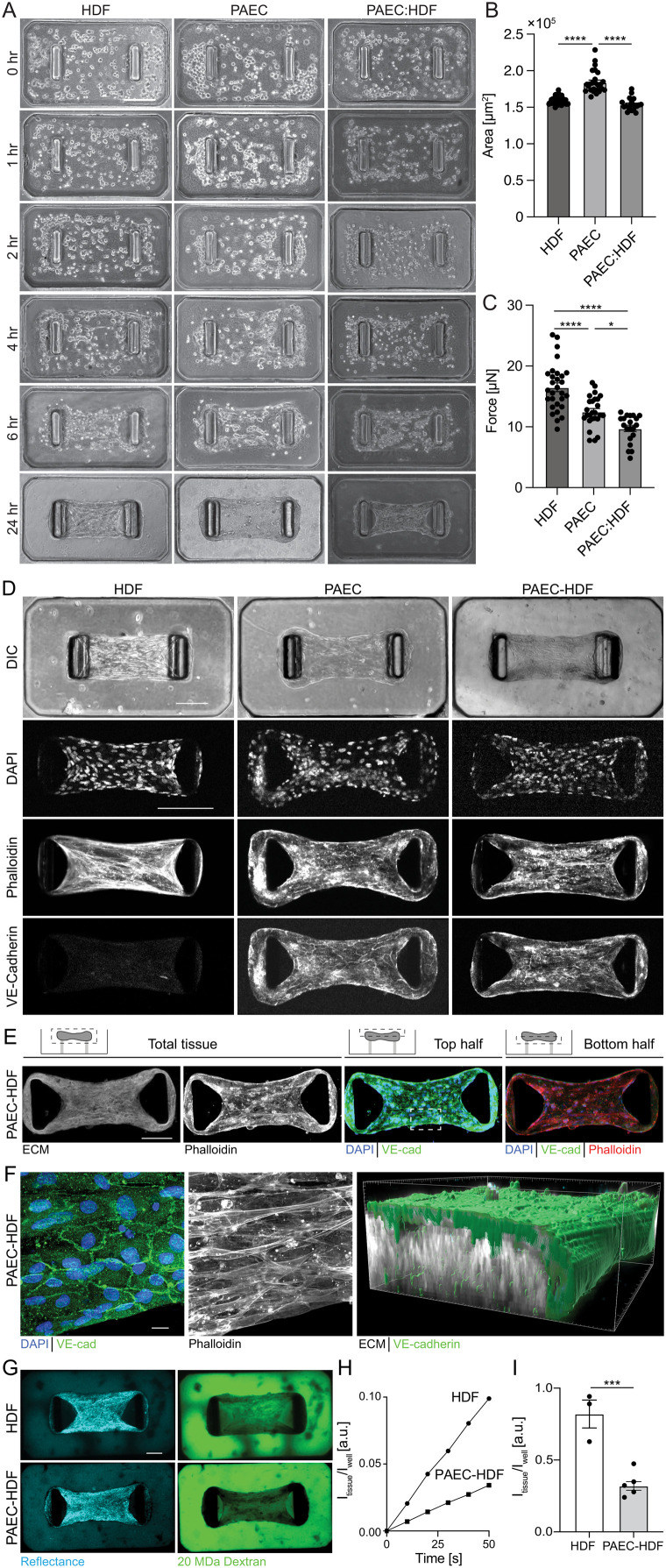
Characterization of pulmonary arterial endothelial microtissue formation, force, area, and organization. (A) Representative phase-contrast images of microtissue formation time course of HDF, PAEC, and PAEC : HDF (4 : 1) seeded at 5 × 10^5^ cells per mL slurry (scale bar 0.24 mm). Quantification of HDF, PAEC, and PAEC : HDF (4 : 1) microtissue (B) projected area and (C) contractile force at 24 h after seeding. (D) Confocal maximum intensity projections of HDF, PAEC, and PAEC : HDF (4 : 1) microtissues (scale bar 0.24 mm). (E) Confocal maximum intensity projections for whole microtissues, top half, and bottom half as indicated by schematic of PAEC : HDF microtissues (scale bar 200 μm). (F) Magnified confocal maximum intensity projection of top slices of microtissue area indicated in (E) and 3D reconstruction showing the spatial organization of VE-cadherin-positive monolayer at the surface of the tissue (scale bar 15 μm). (G) Confocal slices of HDF *versus* PAEC : HDF (4 : 1) microtissues 1 min after adding 20 MDa dextran. Reflectance images used to find the median slice of each microtissue (scale bar 100 μm). (H) Fluorescence intensity of dextran in the center of the microtissue normalized by intensity in the well outside the tissue as a function of time. (I) Normalized fluorescent intensity measured in individual tissues 3 min after adding 20 MDa dextran. *p < 0.05, **p < 0.01, ***p < 0.001, ***p < 0.0001 as determined by one-way ANOVA, with *n* ≥ 3 microtissues. All plots mean ± S.E.M. and each data point indicating an individual microtissue.

To determine feasibility of introducing a functional endothelium into the SMUG model, we first seeded SMUGs with PAECs with and without HDFs (Fig. 3A). We found that while PAECs did contract into microtissues, the inclusion of HDFs resulted in more compact microtissues (Fig. 3B). Interestingly, HDFs reduced the baseline contractile force generated by microtissues (Fig. 3C) and resulted in forces about half that of PASMC : HDF microtissues (Fig. 1F). The presence of PAECs with HDFs resulted in a phalloidin distribution with less apparent stress-fiber formation than HDFs alone, and VE-cadherin staining demonstrated a cobblestone pattern consistent with adherens junction formation (Fig. 3D). Interestingly, the VE-cadherin-positive cells localized to the top surface of microtissues (Fig. 3E), and high-resolution confocal imaging demonstrated that the PAECs formed a monolayer at the surface of the microtissue (Fig. 3F). To test the functional consequences of the PAEC monolayer, we added fluorescent dextran to the media and performed time-lapse confocal imaging to quantify the dynamics of dextran diffusion. We found that dextran diffused more slowly into PAEC : HDF microtissues compared to HDF-only microtissues (Fig. 3G–I), suggesting that the endothelial monolayer presents a functional diffusive barrier.

### 2.5 Pulmonary arterial SMUGs demonstrate endothelial-dependent contractility

**Fig. 4:**
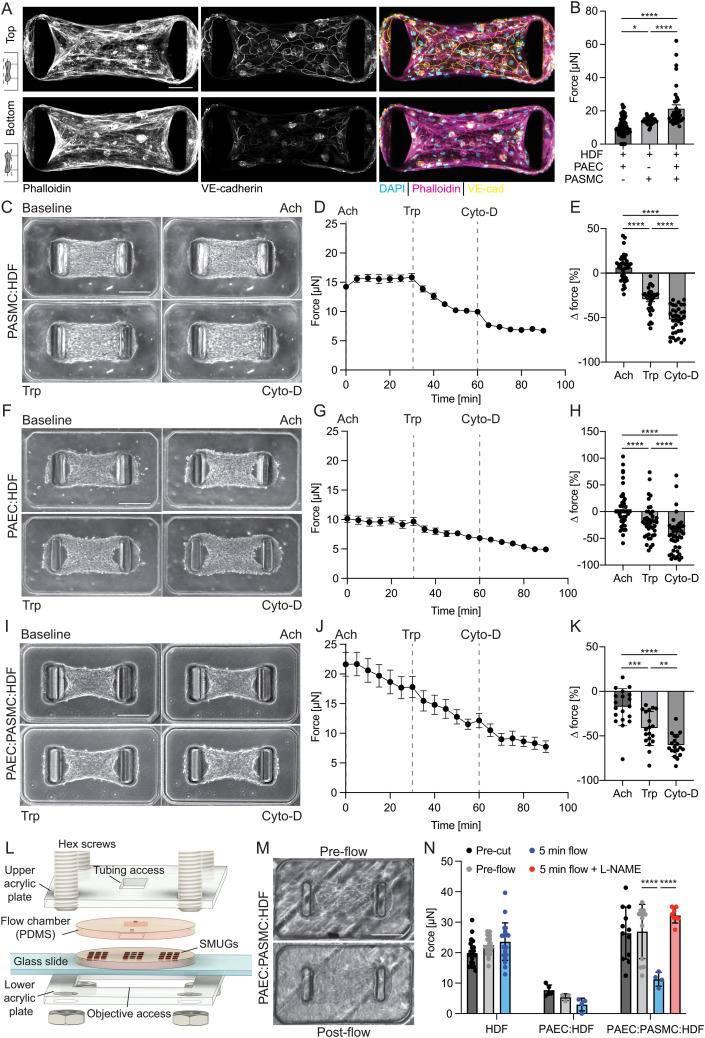
Dynamic vasorelaxation of PA-SMUGs in response to drug treatment and hemodynamic flow. (A) Maximum intensity confocal projections for top half and bottom half of PAEC : PASMC : HDF (5 : 4 : 1) microtissues (PA-SMUGs) as indicated in schematic. (B) Baseline contractile force for duo-culture and PA-SMUGs 24 h after seeding. (C) Representative phase-contrast images of PASMC : HDF (4 : 1) microtissues at baseline and after sequential acetylcholine (Ach), treprostinil (Trp), and cytochalasin-D (Cyto-D) treatments. (D) Dynamic force measurements of PASMC : HDF (4 : 1) microtissues throughout drug treatments at timepoints indicated on graph. (E) Changes in contractile force of PASMC : HDF (4 : 1) microtissues after 30 min drug treatments normalized to baseline contraction values prior to drug treatment (negative values indicate microtissue relaxation). (F) Representative phase-contrast images of PAEC : HDF (4 : 1) microtissues at baseline and after drug treatments as in (C). (G) Dynamic force of PAEC : HDF (4 : 1) microtissues in response to drug treatment. (H) Changes in contractile force of PAEC : HDF (4 : 1) microtissues after 30 min drug treatments. (I) Representative images of PAEC : PASMC : HDF (5 : 4 : 1) microtissues after sequential drug treatment as in (C) (scale bar 0.24 mm). (J) Dynamic force of PAEC : PASMC : HDF (5 : 4 : 1) microtissues throughout drug treatments at indicated timepoints. (K) Changes in contractile force of PAEC : PASMC : HDF (5 : 4 : 1) microtissues after 30 min drug treatments. (L) Schematic representation of flow chamber setup. (M) Representative phase contrast images of PA-SMUGs before and after application of flow to induce 8 dyne cm^−2^ shear stress at the microtissue surface for 5 min. (N) Contractile force of PA-SMUGs and control microtissues with seeded with HDF or PAEC : HDF. Images were acquired prior to removing PA-SMUGs from dish used for seeding (pre-cut), after device assembly and prior to application of flow (pre-flow), and after 5 min of flow with and without L-NAME for the triculture condition (for ****p < 0.001 as determined by *t*-test). For all images, scale bar 0.24 mm. For static experiments, all plots are mean ± S.E.M. from *n* ≥ 36 microtissues, with individual datapoints referring to individual microtissues. *p < 0.05, **p < 0.001, ****p < 0.0001 as determined by one-way ANOVA.

Having demonstrated the ability to quantify function of PASMCs through measurements of contractility and PAECs through measurements of barrier function, we next sought to establish a tri-culture model to recapitulate a pulmonary artery-on-chip. We seeded PASMCs, PAECs, and HDFs in devices to form pulmonary artery (PA-) SMUGs (Fig. S4 and Video S1). To evaluate whether PAECs form a monolayer at the upper surface of microtissues in the tri-culture PA-SMUG microtissues, we fixed microtissues and immunostained for VE-cadherin. Consistent with the PAEC : HDF microtissues, we found that PAECs formed a monolayer with adherens junctions at the top surface of PA-SMUG microtissues (Fig. 4A). Live cell imaging demonstrates that PAEC and PASMC migrate along parallel tracks at similar migration speeds during tissue compaction, suggesting that the segregation of these cell types occurs after initial tissue assembly (Fig. S5 and Video S2). We also found that incorporation of HDFs was necessary for tissue formation (Fig. S6A–C). We compared tri-culture PA-SMUGs with duo-culture of PASMC and PAEC with HDFs (all microtissues seeded at 5 × 10^5^ cells per mL) and found that PA-SMUGs generated higher magnitude baseline force than either HDF : PAEC or HDF : PASMC duo-culture microtissues (Fig. 4B), and that this difference in force was not due to the number of cells per tissue (Fig. S6D).

To determine the functional consequences of the co-culture and tri-culture models, we allowed microtissues to assemble and contract for 24 h before treatment with 10 μM acetylcholine, treprostinil, and cytochalasin-D. Consistent with reports on arteries denuded of endothelial cells,^43^ in response to acetylcholine treatment, we saw a mild contraction of PASMC : HDF microtissues (Fig. 4C–E) with little effect on PAEC : HDF microtissues (Fig. 4F–H), and relaxation in triculture PA-SMUG microtissues (Fig. 4I–K). All three microtissue types relaxed in response to treatment with treprostinil, with no observable difference in relative magnitude or kinetics of relaxation between PASMC : HDF and PAEC : PASMC : HDF microtissues (Fig. 4C and I and S7A). Furthermore, there was no significant difference in relaxation due to treprostinil between PASMC : HDF and PAEC : PASMC : HDF microtissues (Fig. S7B), and relaxation was not due to the addition of DMSO load control (Fig. S7C). Interestingly, Treprostinil had differing effects in donor PASMC : HDF microtissues derived from donor 150 and donor L147 (Fig. S8), which were characterized by the lowest and highest magnitudes of baseline contraction, respectively (Fig. 2). Despite the baseline contraction values, microtissues with PASMCs from donor L147 did not relax in response to treprostinil (Fig. S8). Together with the response to drug treatments, the cellular distribution suggests that PA-SMUGs serve as a functional reductionist model of pulmonary arterial tissue.

To test endothelial-mediated flow-dependent vasodilation within the SMUG system, we fabricated a microfluidic device to apply flow to SMUGs after seeding (Fig. 4L). Using a syringe pump, we applied flow to impart 8 dyne cm^−2^ wall shear stress at the microtissue surface. In PAEC : PASMC : HDF triculture tissues, application of shear stress induced relaxation (Fig. 4M and N). No such relaxation was observed in HDF or PAEC : HDF control samples, suggesting that flow-induced relaxation requires PAECs and PASMCs. In donors, flow-mediated dilation is driven by release of NO from the endothelium,^44^ and to determine whether NO release mediates flow-mediated relaxation in PA-SMUGs, we repeated experiments with *N*-nitro-l-arginine methylester (l-NAME), an inhibitor of nitric oxide synthetase (NOS), and found that relaxation was attenuated (Fig. 4N), further suggesting that flow-mediated nitric oxide release by PAECs drives the observed relaxation.

## 3. Discussion

PAH progression involves dysregulation of vascular tone,^8,9^ yet the development of novel effective treatments is hindered by the lack of humanized assays that recapitulate endothelial-dependent vasodilation. In this work, we leveraged microfabrication to develop multiplexed microtissue contractility gauges (Fig. 1) that are seeded with primary human PASMCs and PAECs. We found that the resultant microtissues generate micronewton magnitude forces at baseline and dilate in response to native vasomodulators and treprostinil, a synthetic prostacyclin analog that is a standard of care for treating PAH subpopulations^45^ (Fig. 4). Current models of PAH lack the throughput necessary for therapeutic screening and rely on endpoint contraction to quantify vasomodulation. Thus, the platform described here, which allows for high-throughput and dynamic quantification of vasoconstriction and vasodilation in donor-derived arterial tissues, represents a significant advancement in PAH disease modeling *in vitro*.

Interestingly, we found that cells seeded into the PA-SMUG platform self-assemble into contractile units with a distinct endothelial monolayer and basal smooth muscle layer (Fig. 3 and 4), reminiscent of the cellular distribution of the arterial intima and tunica media. While this is the first report of self-assembly of PAECs and PASMCs in contractile microtissues, the non-uniform cell distribution is consistent with previous reports from a microtissue wound healing model in which fibroblasts migrated into a wound as a planar sheet at the top of the tissue.^37^ Live cell imaging demonstrates that the self-assembly of the PAEC monolayer occurs after initial tissue compaction (Fig. S5 and Video S2). While further investigation is necessary to determine how ECs assemble at the top surface of the microtissue, ECs migrate at higher speeds on 2D substrates than in 3D,^46^ and VE-cadherin engagement is known to inhibit cell migration.^47^ Thus, we hypothesize that after tissue compaction, ECs migrate to the surface of the tissue where they engage neighboring cells *via* VE-cadherin and establish a monolayer. Additionally, we observe that PA-SMUGs seeded with PAECs generate higher magnitude forces than PASMC-HDF co-culture tissues (Fig. 4B), which is surprising given that PAECs generate lower magnitude forces than HDFs (Fig. 3C). We hypothesize the difference in force generation is due to the microstructure of the tissues, as PA-SMUG triculture tissues are characterized by increased actin bundling along the longitudinal microtissue axis as compared to tissues without PAECs (Fig. 1G and 4A), and cell and tissue alignment are known to regulate force transmission and total force applied to cantilevers in microtissue models.^48^ In future experiments, we plan to conduct live cell imaging of the cytoskeleton of each cell type for longitudinal studies of tissue assembly, cytoskeletal organization, and resultant force magnitude. Interestingly, we observe mild contraction in PASMC-HDF co-culture microtissues and relaxation in triculture PA-SMUGs. These observations are consistent with previous reports from a rabbit model where treatment of acetylcholine in excised aortic strips caused relaxation, but when the strips were denuded of the endothelium, acetylcholine treatment led to mild contraction.^43^

Furthermore, while the PAEC and PASMC cell distribution is reminiscent of the artery, with the EC apical surface exposed to fluid and the basal surface exposed to ECM with subluminal SMCs, the cells are separated by a collagen hydrogel with embedded fibroblasts (Fig. 3 and 4), a key cell type in the adventitia. Thus, from an arterial anatomy standpoint, the adventitia and media in the microtissues reported here are inverted, which likely reduces juxtracrine signaling such as Notch receptor–ligand interactions that have been shown to be critical regulators of arterial tone.^49^ Furthermore, the lack of continuous coverage of the SMC compartment by ECs allows molecular diffusion around the endothelium and thus does not recapitulate the full transport barrier presented by the endothelium in a native artery. In future work, these structural deficiencies could be addressed through multi-layer seeding approaches^18–20^ or the selective removal of fibroblasts after tissue assembly.^50^ Another challenge in any multicell culture system is differences in media constitution for each cell type, and while we did not see differences in terms of cell number in microtissues seeded with different cells and growth media (Fig. S6D), differences in growth factor concentrations could drive differences in microtissue force generation. Common to all organ-on-chip systems, the development of common and shared media among the constituent cell types would help improve consistency in future iterations.^51^ Despite these challenges, a key benefit of the approach described here is the ease of use and high-throughput microtissue generation from a single seeding event. Furthermore, a hallmark of severe pulmonary hypertension is the formation of a layer of myofibroblasts and ECM between the endothelium and the internal elastic lamina, termed the neointima,^12^ and thus the microtissues described here could serve as a useful model for understanding and intervening in neointima formation in PAH progression.

While it is broadly understood that PAH involves endothelial dysfunction,^10,11^ there remains a question as to the relative contributions of the endothelium and smooth muscle layer in the dysregulation of vascular tone. We hypothesized that SMCs from patient donors would demonstrate elevated contractility compared to controls. However, we found that averaged over the cohort, patient cells had same level of contractility as healthy controls (Fig. 2). We did observe interpatient variability in microtissue formation, contractile force, and cellular architecture (Fig. 2), suggesting that donor-specific PA-SMUGs could be used to investigate mechanisms that lead to varied outcomes in treatment and for patient risk stratification to optimize care. Indeed, preliminary studies indicate donor variability in the response to treprostinil treatment (Fig. S8). While it is difficult to contextualize these results without more detail on patient history and disease presentation, clinical data has demonstrated heterogeneity in the response of PAH patients to treprostinil.^52^ Furthermore, the severity of disease and comorbidities for individual donors are unknown and could underlie the heterogeneity observed between samples. An advantage of the PA-SMUG approach is the relatively small number of cells required to assemble contractile tissues, which will allow seeding of tissues and sequencing studies, such as bulk RNAseq, from a single patient-specific culture. Such an approach, particularly when integrated with automated culture technology such as liquid handling systems, will allow correlations between gene expression profiles, contractility, and drug responses to provide mechanistic insight into the molecular underpinnings of donor variability.

The relative contributions of PAEC *vs.* PASMC dysfunction in PAH progression are also thought to be a function of disease progression, and the molecular and cellular PAEC alterations in early stage PAH precede muscularization of pulmonary arteries in patients and animal models.^12,13^ Additionally, it is thought that EC-dependent modulation of SMC proliferation and NO release by ECs, rather than basal SMC contractility, underlie disease progression.^13^ In future work, PA-SMUGs will be used to combinatorially assemble tissues with healthy and IPAH donor-derived PAECs in addition to PASMCs, allowing for investigation of the mechanisms that lead to PA dysfunction, which drives hypertension in a patient-specific manner. Integrating these patient-specific PA-SMUGs with NO perturbations could allow prognostic and mechanistic screening of disease severity *in vitro*, informed by the use of inhaled NO as a clinical prognostic indicator.^53^

While the results presented here address shortcomings of previous approaches, there are several limitations to the PA-SMUGs that fail to recapitulate key features of arterial physiology. The studies with donor cells described here were completed in static conditions, but hemodynamic signals are known to be key regulators of arterial tone.^44^ Informed by recent work for measuring clot contraction under shear stress,^54^ we fabricated microfluidic flow chambers that can be affixed to PA-SMUGs after microtissue compaction (Fig. 4). Using these chambers, we observed a PAEC- and PASMC-dependent relaxation with the application of 8 dyne cm^−2^ shear stress to the tissue surface, and importantly, this relaxation was attenuated with the inhibition of NOS by l-NAME (Fig. 4). This approach represents an important step toward the development of an *in vitro* flow-mediated dilation assay, which is a clinical standard for endothelial dysfunction in multiple disease states.^55^ Similarly, the PA-SMUG devices do not explicitly recapitulate blood pressure or the circumferential stresses that develop in the arterial wall to balance blood pressure.^56^ Tissue engineered blood vessels have been developed that allow modulation of luminal pressure,^57^ but these models are technically challenging and low-throughput. Future adaptations of the PA-SMUG model could include magnetic actuators^58^ to enable active force application as a surrogate for blood pressure. Recent developments in dynamic light processing (DLP) 3D printing have allowed rapid prototyping of microtissue models, which will enable rapid prototyping to optimize microwell geometry and micropillar stiffness to maximize dynamic range of tissue-specific SMUG models.^59^ These prototyping methods will also allow more complex cap geometries that can constrain microtissues on more compliant micropillars to improve dynamic range of force calculations. Another limitation of the approach reported here is the relatively short time scales of culture (72 h) to model PAH, due in part to tissues popping off deflected microposts. Previous work has demonstrated that milling pillar structures on the millimeter-scale allowed sustained culture of cardiac microtissues for 4 weeks,^60^ and the work reported here establishes the baseline design parameters for future systems that can sustain longer-term culture to be more suitable for chronic disease modeling. The current iteration of PA-SMUGs also lacks an active immune component, and inflammation and immunity have been shown to be critical in the pathogenesis of PAH.^61^ Future studies will integrate patient blood and serum^62^ to allow further interrogation of circulating immune cell activation and signaling in PAH progression.

In summary, this study provides a new model to examine the cellular contributions to PA dysfunction in the progression of pulmonary hypertension. By demonstrating functional microtissues that provide calibrated quantification of EC-dependent vasorelaxation, this microphysiologic 3D pulmonary arterial model may become a valuable tool to investigate mechanisms and to screen potential interventions to treat patients with this devastating disease.

## 4. Methods

### SMUG mold fabrication

SMUGs were fabricated using multilayer photolithography. Silicon wafers (100 mm single side polish, test grade, University Wafer, Boston, MA) were rinsed with 5% (v/v) hydrofluoric acid (Sigma-Aldrich, St. Louis, MO) for 1 min to strip the oxide layer. To improve adhesion of high-aspect ratio structures, wafers were spun with SU-82005 (Kayaku Advanced Materials, Westborough, MA) to a first-layer thickness of 5 μm, soft baked for 2 min at 95 °C, and flood exposed using a mask aligner (MA6/BA6, Suss Microtec, Garching, Germany). Spin rates and exposure parameters can be found in SI Methods (Table S1). Wafers were post-exposure baked for 3 min at 95 °C and cooled to room temperature (RT). Wafers were then spun with SU-82150 (Kayaku Advanced Materials) to create the pillar layer (Fig. S2). The wafers were soft baked for 10 min at 65 °C and 30 minutes at 95 °C, with a ramp of 5 °C min^−1^. During the soft bake, a blocking layer^36^ was mixed from SU-82010 and S-1813 Kayaku advanced materials, volume ratios described in SI methods. After wafers were soft baked and cooled, the blocking layer was spun over the pillar layer and soft baked. After cooling, the pillar and blocking layer were exposed together through a film photomask (Fineline Imaging, Colorado Springs, CO) and a 365 nm UV filter. The exposure time depended on the projected height of the structures and the amount of positive (light-absorbing) photoresist in the blocking layer (Table S1). After cooling, wafers were spun with SU-82050 (Kayaku Advanced Materials) to create the cap layer with dimensions as described in Fig. S2. The wafers were soft baked for 10 min at 65 °C and 30 min at 95 °C. After cooling, the caps were exposed through a film photomask and a 365 nm UV filter, aligned to exposed features using a mask aligner. During this step, the blocking layer partially occludes the pillar layer from the cap exposure, allowing for the creation of a flare at the cap. The wafers were post-exposure baked for 10 min at 65 °C and 30 min at 95 °C. After cooling to RT, the wafers were developed for 15–30 min in SU-8 developer (Kayaku Advanced Chemicals) and were rinsed for 2 min in isopropyl alcohol (IPA, Sigma-Aldrich), and development status was checked with an upright microscope (Nikon Eclipse LV150). The wafers were measured for total height using a profilometer (F50 Thin Film Mapper).

### SMUG soft lithography

To generate SMUGs in individual 15 mm petri dishes (Fisher Scientific, Hampton, NH), multi-step replica molding was used to generate positive polydimethyl siloxane (PDMS, Sylgard 184, Dow, Midland, MI) SMUGs from the positive silicon wafer mold. First, the silicon wafer was treated for 30 s at 30 W using an expanded plasma cleaner (Harrick Plasma, Ithaca, NY). Then, wafers were treated with trichloro(1*H*,1*H*,2*H*,2*H*-perfluorooctyl)silane (Sigma-Aldrich) through overnight vapor deposition in a vacuum chamber. To create a negative mold of the pillars, a “stamp” of the wafer was created. PDMS was mixed at a 10 : 1 ratio of base : crosslinker and was degassed for 1 h in a vacuum chamber. The degassed mixture was poured onto the wafer and was allowed to rest at RT for 30 min for the PDMS to enter the cavities surrounding the pillars, as we found that further degassing could cause bubbles that delaminated the pillars. Wafers with uncured PDMS were then baked at 65 °C overnight. The PDMS stamp was detached from the wafer through careful cutting of the PDMS and application of IPA as the stamp was lifted from the mold. Resulting SMUG negative molds were punched from the overall stamp using a 24 mm arch punch (CS Osborne, Harrison, NJ). The SMUG negative molds were activated with plasma for 30 s at 30 W, and trichloro(1*H*,1*H*,2*H*,2*H*-perfluorooctyl)silane was vapor deposited overnight. To prepare individual devices within 15 mm dishes, 1 g of uncured PDMS was poured into each 15 mm petri dish and was cured for 2 h at 65 °C on a hot plate to create a flat PDMS base for the SMUGs. After cooling, 1 g of uncured PDMS was poured again into each dish, and 0.5 g of PDMS was poured onto each SMUG negative. The SMUG negatives were degassed for 30 min in a vacuum chamber (Fig. S9). PDMS-coated negative stamps were flipped into the petri dishes of uncured PDMS to mold positive SMUGs into each dish. After curing overnight at 65 °C, IPA and a razor blade were used to release the negative stamps. The devices were first washed with a 1 : 1 mixture of IPA and deionized water (DI-H_2_O) to remove excess silane and were washed again with DI-H_2_O. The devices were allowed to dry before use.

### Fabrication and imaging of fluorescent PDMS

A solution was prepared of 0.04% (w/v) of Nile red (Thermo Fisher Scientific) in α-terpineol (Sigma-Aldrich). The solution was sonicated for 1 minute using an SFX150 Sonifier (Branson Ultrasonics, Brookfield, CT), and was centrifuged at 1503 g for 5 min at room temperature to separate undissolved Nile red. The supernatant was collected and transferred to a new tube, and was added at 0.4 mL of solution per 5 g of PDMS base. The base with the dye solution was mixed and then degassed for 30 min before being heated on a hot plate at 210 °C for 40 min to evaporate away the excess α-terpineol. The mass of the PDMS-dye solution was checked before and after heating to ensure evaporation. After cooling the PDMS-dye base, crosslinker was added at a 1 : 10 ratio of crosslinker : final mass of PDMS-dye base. The crosslinker was gently mixed into the base before being degassed for 15 min. Then, the fluorescent PDMS was substituted for plain PDMS in the replica molding protocol previously described. After demolding the stamps, devices were punched out of the dishes and placed on a coverslip for imaging on an Olympus F3000 laser scanning confocal with a 10× U Plan S-Apo, 0.4 numerical aperture (NA) air objective. Devices were placed right-side-up and upside-down on the coverslip to counteract signal loss near the top of the sample, and the two orientations of the images were merged before being reconstructed as a 3D rendering using Imerys software.

### Cell culture

HDFs (ATCC, Manassas, VA) were cultured in 1× low-glucose Dulbecco's modified eagle's medium (DMEM, Gibco, Thermo Fisher Scientific, Waltham, MA) and were used between passages 7–12. PAECs (ATCC) were cultured in microvascular EGM-2 (Lonza, Basel, Switzerland) and were used between passages 5–9. Healthy PASMCs (ATCC) were cultured in SmGm-2 (Lonza) and were used between passages 5–7. Donor PASMCs were obtained from the Pulmonary Hypertension Breakthrough Initiative (PHBI) tissue bank under an approved protocol, and all PASMCs were derived from type III (≤1 mm) pulmonary arteries. All cells were cultured in a humidified incubator at 37 °C and 5% CO_2_. Cells were grown to a confluency of 80–90% before being passaged with 0.05% (w/v) trypsin-EDTA (Thermo Fisher Scientific).

### SMUG seeding

SMUGs in dishes were sterilized with 70% v/v ethanol in DI-H_2_O for 20 min before being rinsed twice with DI-H_2_O. Then, 2 mL of 0.5% w/v pluronic F127 (Sigma-Aldrich) in DI-H_2_O was applied to each device, and devices were centrifuged at 200 g for 2 min, incubated for 30 min at RT, and rinsed twice with DI-H_2_O. Collagen hydrogels were prepared as described previously.^63^ Briefly, reconstitution buffer (RB) was prepared by dissolving 1.2 g of NaHCO_3_ and 4.8 g of HEPES in 50 mL of DI-H_2_O, and sterile filtering. Low- and high-concentration type I collagen from rat tail in acetic acid (Dow) was mixed to a working concentration of 5 mg mL^−1^ collagen I in acetic acid. Collagen I working stocks were diluted in equal volume of 10× DMEM (with 4500 mg L^−1^ glucose and l-glutamine, without sodium bicarbonate, Sigma-Aldrich) and RB (1 : 10 v/v in collagen I working stock), and growth media (1 : 10 v/v in total hydrogel volume) and PBS were added to bring the total slurry volume to 500 μL for a final collagen concentration of 2.5 mg mL^−1^ total solution. Prior to each cell seeding, test slurries were used to titrate solution pH to 7.5–8 using 1 N NaOH (Sigma-Aldrich) and to measure polymerization time. After NaOH volumes were determined from test slurries, cells were lifted with 0.05% w/v trypsin-EDTA, centrifuged for 5 min at 200 g, and resuspended in growth medium. For HDF validation experiments, HDFs were suspended to 10 × 10^6^ cells per mL. For all other experiments, cells were resuspended to 5 × 10^6^ cells per mL. For duoculture experiments, the cells were combined at an 4 : 1 ratio as described previously^41^ (PASMC : HDF and PAEC : HDF). For triculture experiments, the cells were combined at a 40 : 50 : 10 ratio (PASMC : PAEC : HDF). The cells were incorporated into the gel at a 1 : 10 v/v ratio to the total gel slurry (replacing the growth medium in the test slurry), and 900 μL of the cell-laden slurry was pipetted into each device. The devices were centrifuged at 200 g for 1 min at 4 °C. After centrifugation, excess slurry was removed from the device using a vacuum aspirator and gel-loading pipette tip. Devices were then incubated at 37 °C for 10–20 min, depending on polymerization kinetics of test slurries. After polymerization, 1 mL of growth medium was slowly added so as not to disturb the microtissues. For devices including multiple cell types, the appropriate growth medias were combined according to the cell ratios within the device. The devices were allowed to form microtissues for 24 h unless otherwise stated.

### Dynamic SMUG imaging

Cell-seeded SMUG devices were maintained in the incubator for 24 h after seeding and were placed into a heated and CO_2_ supplied closed chamber (Tokai Hit) and imaged *via* widefield microscopy. Widefield imaging was performed on an Olympus IX83 microscope with a 10× U Plan FL, 0.3 and acquired on an Orca-Flash 4.0 LT (Hamamatsu, Bridgewater, NJ). Devices were serum starved in 1 mL of EBM-2 (Lonza) supplemented with 0.2% FBS (Avantor) during thermal equilibration on the microscope stage. After 30 min, devices were imaged for baseline SMUG deflection. Drug treatments were suspended to a final concentration of 20 μM in serum starvation media. First, half of the existing media on the devices were aspirated, and 500 μL of acetylcholine-treated media was applied to each device, leading to a final drug concentration of 10 μM. Images of all SMUGs were captured every 5 min for 30 min. Subsequently, half of the media was replaced with the 20 μM treprostonil treatment and devices were similarly imaged. Finally, half of the media was replaced with 20 μM of cytochalasin-D treatment and devices were similarly imaged.

### Flow chamber fabrication and assembly

The flow chamber was designed in Adobe Illustrator and fabricated using laser-cutting of 1.5 mm thick clear acrylic. The bottom plate of the chamber was designed to fit a 25 mm wide glass slide and included a 25 mm square window allowing for imaging with an inverted microscope (Fig. 4L). The flow chamber was designed using Adobe Illustrator and fabricated with a Bambu Lab (Shenzhen, China) X1C 3D printer with polylactic acid (PLA) filament. The flow chamber is a 24 mm diameter circle pad with a 6 mm wide × 20 mm long × 200 μm deep rectangular channel (PDMS flow chamber in Fig. 4L). The flow chamber was molded from the 3D printed mold using PDMS using soft lithography. The top acrylic plate included a window matching the flow chamber dimensions to clamp the chamber down and provide tubing access (upper acrylic plate in Fig. 4L). Through holes 3 mm in diameter were included on the top and bottom acrylic plates to allow hex screws to align the plates. The screws were tightened using hex nuts, clamping the system shut to prevent leakage. The flow chamber was glued onto the top acrylic plate using a thin layer of PDMS. After cell seeding, the SMUG devices were cut and mounted on a glass slide. The mounted slide and chamber components were all submerged in pre-warmed media while the flow fixture was quickly assembled and tightened to clamp the sample and flow chamber together.

### Hemodynamic flow experiments

SMUG tissues were imaged within the original wells before starting the experiment (pre-cut condition in Fig. 4N). The SMUGs were removed from the wells and clamped into the flow chamber as described above. The assembly was submerged in warm media to preserve the integrity of the microtissues and to avoid bubbles. The media was formulated per cell type as described in the cell seeding section of the methods. After assembly in the flow chamber, the SMUGs were allowed to equilibrate for 15 min in the incubator before the microtissues were re-imaged (pre-flow condition in Fig. 4M and N). The assembly was flushed with media and submerged in a 37 °C water bath to hold the inlet and outlet at constant pressures and to maintain temperature. A pipette tip was marked 2 cm above the water bath line to produce the appropriate hydrostatic pressure to apply 8 dyne cm^−2^ flow, as determined from the channel geometry and rectangular Poiseuille flow. The filled tip was inserted into the device, and a syringe pump was run at 2 mL min^−1^ to maintain a constant hydrostatic pressure, flow rate, and associated wall shear stress. Tissues were imaged after 5 min of flow (post-flow condition in Fig. 4M and N). To inhibit nitric oxide synthase, 70 μM *N*-nitro-l-arginine methylester (l-NAME, Selleck Chemicals, Houston, TX, USA) was added into the media during flow chamber assembly and while applying flow. Images were acquired on an Olympus IX83 widefield microscope with a 4× objective and analyzed as described below.

### Cell staining and visualization

Mono- and duo-culture microtissues were fixed with warm 4% paraformaldehyde (PFA) in PBS containing calcium and magnesium (PBS++) for 20 min at 37 °C. Microtissues were then permeabilized using 0.1% Triton-X-100 (Millipore Sigma-Aldrich) at RT for 10 min. Tri-culture microtissues were perm-fixed^64^ by first incubating with a solution of 1% PFA in PBS++ and 0.05% Triton-X-100 for 90 s at 37 °C before fixing with 4% PFA for 15 min at 37 °C. Fixed devices were washed and stored with PBS++. Primary antibodies against VE-cadherin (1 : 200, v/v, Santa Cruz Biotechnology, Dallas, TX) and αSMA (1 : 200, v/v, Abcam, Cambridge, UK) were diluted in blocking buffer (2% w/v BSA in PBS++) and applied overnight on a laboratory rocker at 4 °C. After primary conjugation, devices were rinsed 3 times with blocking buffer. Secondary antibodies were diluted in blocking buffer (1 : 500, v/v, goat anti-mouse AlexaFluor 488 and goat-anti mouse AlexaFluor 594, Thermo Fisher Scientific) and applied to devices on a laboratory rocker for at least 5 h at 4 °C. Devices were washed 3 times with blocking buffer. Then, DAPI (1.5 : 1000, v/v, Thermo Fisher Scientific) and rhodamine phalloidin (1 : 250, v/v, Thermo Fisher Scientific) were diluted in blocking buffer and applied to devices on a laboratory rocker at RT for 20 min and 1 h, respectively. Devices were washed 3 times with blocking buffer and imaged on an Olympus F3000 laser scanning confocal with a 10× U Plan S-Apo, 0.4 numerical aperture (NA) air objective or a 30× U Plan S-Apo N 1.05 NA silicone oil immersion objective. Widefield imaging was performed on an Olympus IX83 microscope with a 10× U Plan FL, 0.3, and optical microscopy of silicon wafers was performed on an Olympus SZX10 stereo microscope.

### Quantification

Quantification of micropillar deflection was performed using Fiji ImageJ software. Images were scaled to known dimensions of the microwell size, and deflection measurements were taken from the edge of the micropost to the edge of the cap. Deflections of the left and right pillar were averaged, and average deflection in microns was converted to force using estimated stiffness coefficients for the micropillars, which was determined by their geometry and the Young's modulus of PDMS.^36^ Plotting and statistical analysis was done in Prism 10.

## Author contributions

W. J. P., A. S., and R. P. designed the experiments. A. S., R. P., M. R., and C. P. W. conducted the experiments, and W. J. P., A. S., R. P., R. N. S., and M. R. analyzed the data. W. J. P. and A. S. wrote the manuscript. All authors reviewed and edited the manuscript.

## Conflicts of interest

W. J. P. receives research support from United Therapeutics.

## Supplementary Material

LC-025-D5LC00474H-s001

LC-025-D5LC00474H-s002

LC-025-D5LC00474H-s003

LC-025-D5LC00474H-s004

## Data Availability

The data that support the findings of this study are available from the corresponding author upon reasonable request. Supplementary information: including supplementary data and videos and detailed methods for SMUG fabrication. See DOI: https://doi.org/10.1039/d5lc00474h.
