# Supplementary material for: Endothelial-smooth muscle microgauges for modeling pulmonary arterial vasoregulation
Source: Lab Chip. 2025 Oct 13;25(23):6177–90. doi: 10.1039/d5lc00474h (PMC12538385; doi:10.1039/d5lc00474h)
Supplement: LC-025-D5LC00474H-s004 [file LC-025-D5LC00474H-s004.pdf]

Supplementary Information (SI) for Lab on a Chip.
This journal is © The Royal Society of Chemistry 2025

1. Supplementary Results

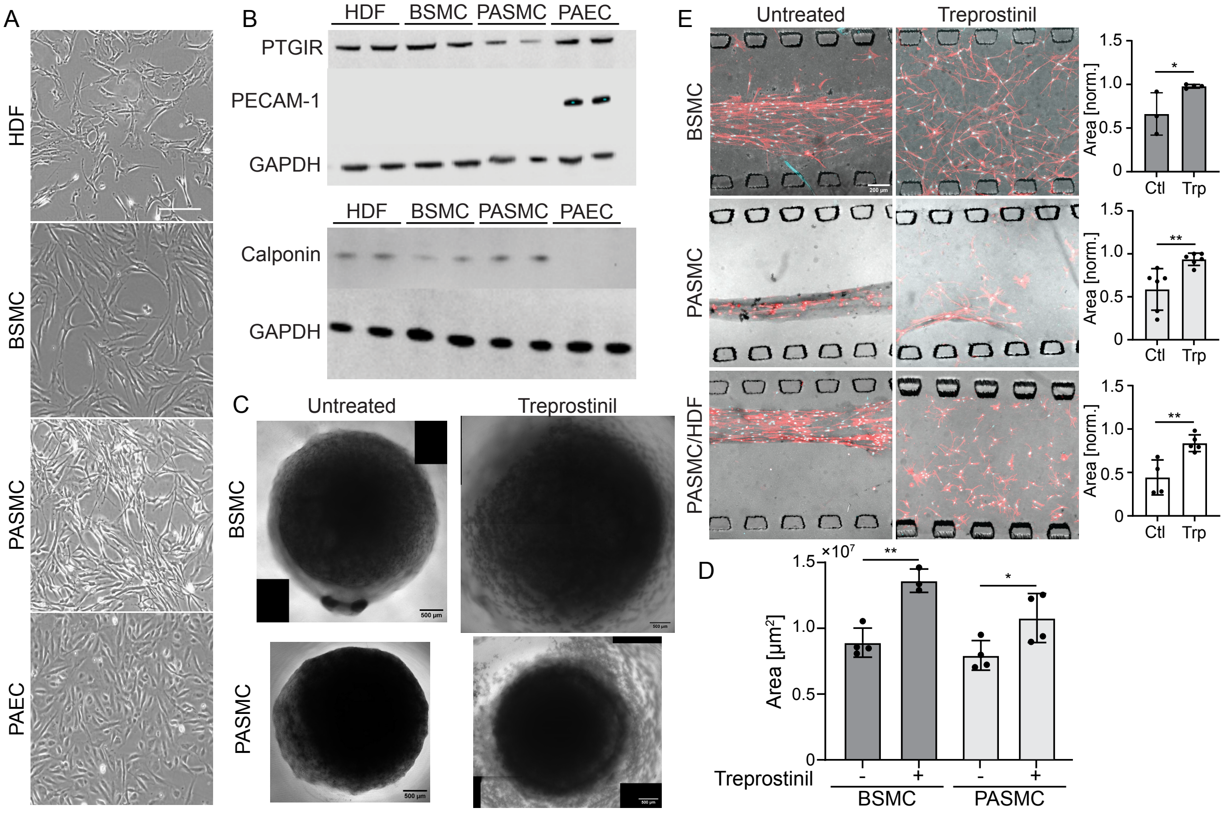

Figure S1. Characterization of baseline contractility for HDF, BSMC, PASMC, and PAECs. A) Representative phase contrast images of HDF, BSMC, PASMC, and PAEC cells in culture (scale bar 50 μm). B) Western blot of PGIR, PECAM-1, and Calponin. C) Representative 4x DIC images of untreated and 10 μM Treprostinil treated BSMC and PASMC embedded in 2.5 mg/mL collagen hydrogels, and D) quantification of hydrogel area (n=4, one-way ANOVA with Bonferroni's post-test, scale bar 500 μm). E) Representative images and quantification of hydrogel area for BSMC, PASMC and 80:20 PASMC:HDF after 18 hrs of culture in microfluidic devices with and without 10 μM Treprostinil (blue – DAPI, red – phalloidin; n = 3-5; scale bar 200 μm; two-tailed Student's t-test; for all figures, * $p < 0.05$, ** $p < 0.01$).

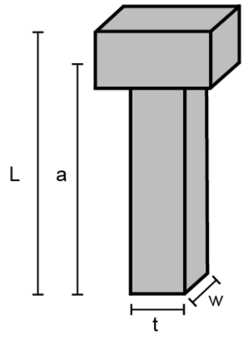

$SMUG_{1.45}$	
w	230 μm
t	62 μm
E	$1.61\ \frac{\mu N}{\mu m^2}$
a	235 μm
L	265 μm

$SMUG_{0.52}$	
w	227 μm
t	35 μm
E	$1.61\ \frac{\mu N}{\mu m^2}$
a	186 μm
L	207 μm

Figure S2. Schematic and tables showing parameters and measured values for SMUG cantilever stiffness calculations. *E* is the stiffness of PDMS from Ref. 36.

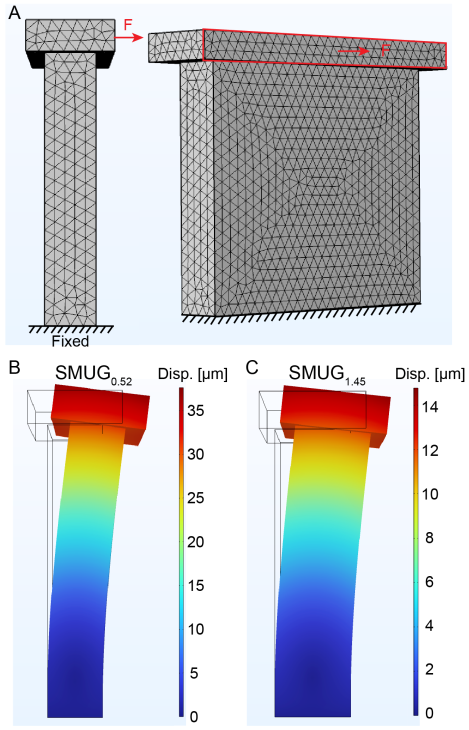

Figure S3. Finite element modeling of micropillars to determine stiffness. (A) Mesh for finite element simulation, and a force of 20 μN was applied at the cantilever cap surface, and displacements were modeled using linear elasticity for SMUGs (B) with a nominal stiffness of 0.52 μN/μm and (C) with a nominal stiffness of 1.45 μN/μm.

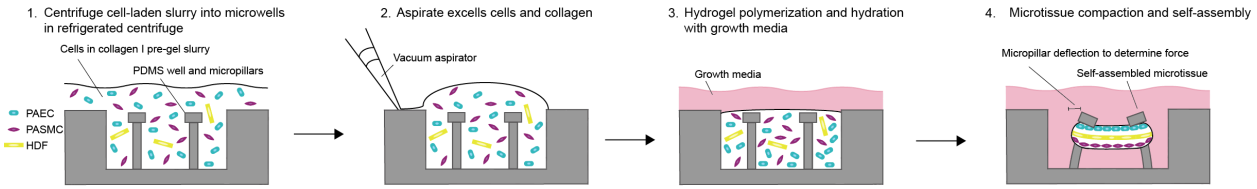

Figure S4. Seeding PA-SMUG microtissues. General schematic that illustrates the process of seeding devices with PAEC, PASMC, and HDF to form PA-SMUG microtissues.

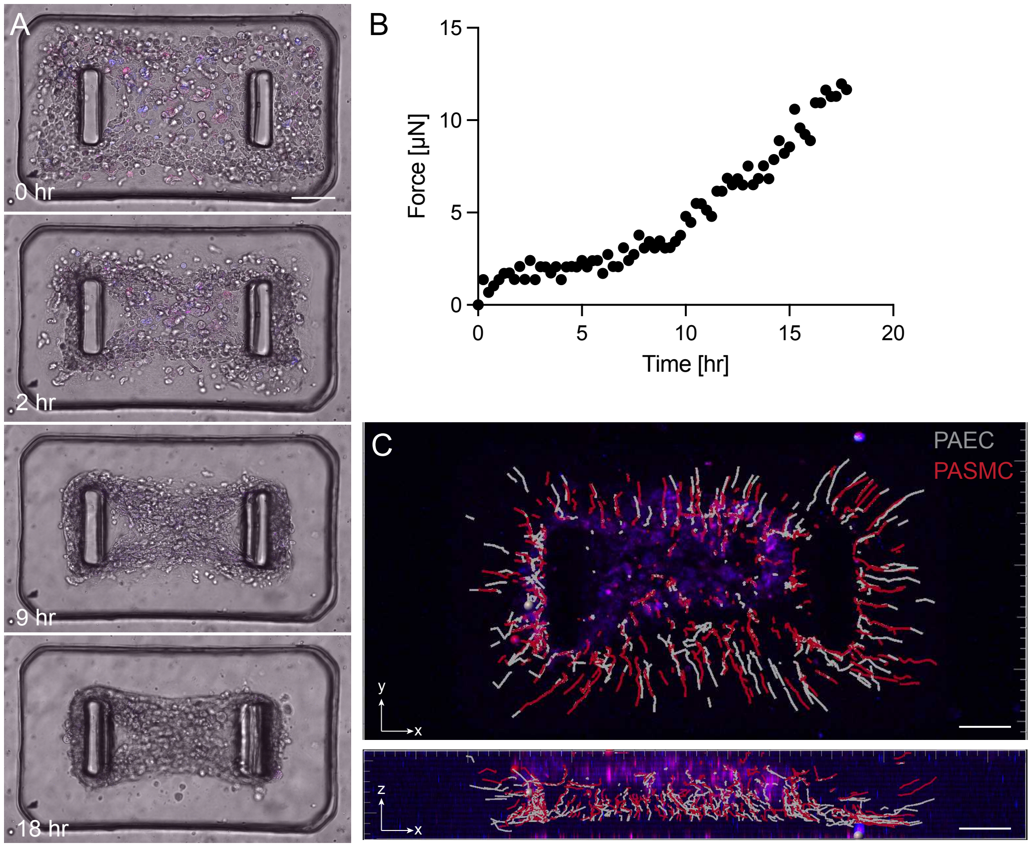

Figure S5. Assembly of tri-culture SMUGs. A) DIC images from 18hr timelapse showing tissue assembly of SMUGs seeded with PAEC, PASMC, and HDFs. B) Quantification of force as a function of time for microtissue shown in (A). Full video is included in Supplementary Information. C) Tracks of individual cell types labeled with SpyFast cytoskeletal dyes (red tracks are PASMC, grey tracks PAECs). Full video is included in Supplementary Information. Scale bars 100 μm.

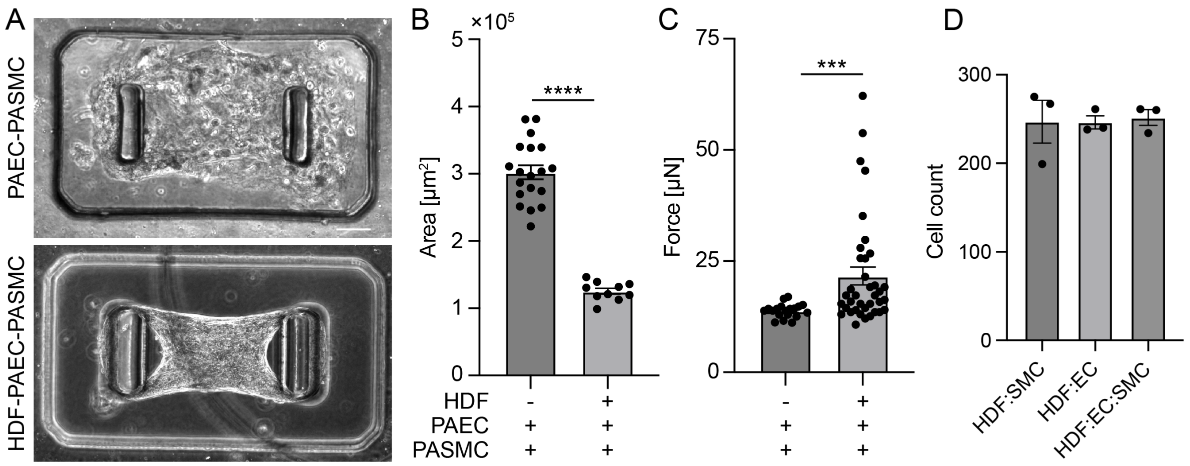

Figure S6. Assembly of PA-SMUGs with and without fibroblasts. (A) Phase contrast images of microtissues assembled with PASMC and PAEC with and without fibroblasts (scale bar 100 μm). (B) Quantification of tissue area and (C) force for tissues formed with and without fibroblasts (****p<0.0001, ***p<0.001 as determined by t-test. All plots mean ± S.E.M and each data point indicating an individual microtissue). (D) Total cell counts as determined by endpoint DAPI staining of microtissues 24 hrs after seeding.

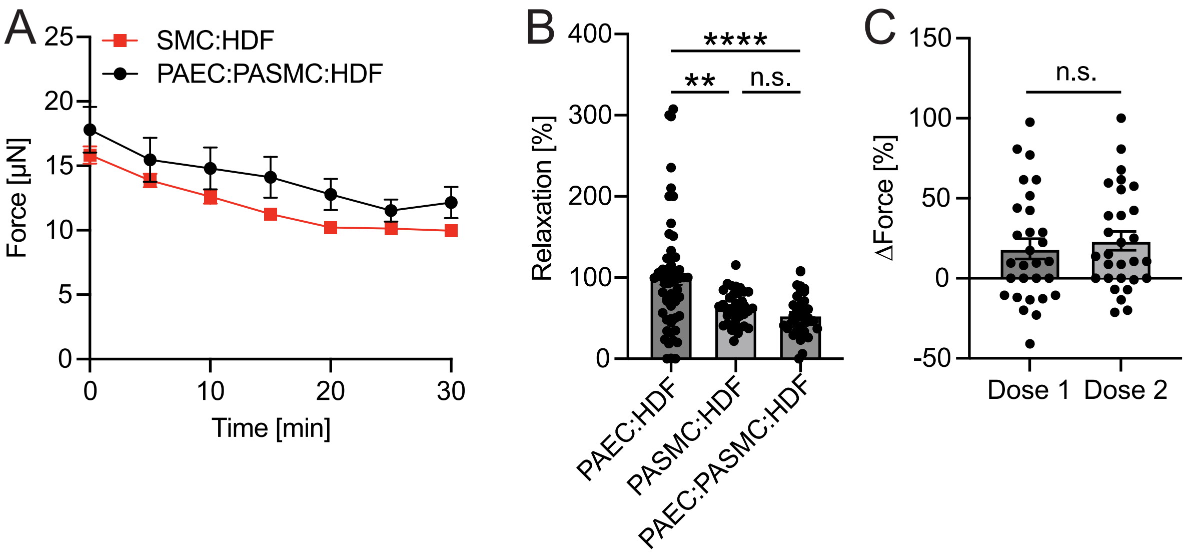

Figure S7. Cell type-specific effects of Treprostinil treatment and validation of vector control. A) Comparison of microtissue dynamics between the PASMC duo-culture and the tri-culture conditions in response to Treprostinil treatment. B) Relative relaxation of microtissues in response to Treprostinil treatment. Relaxation % is calculated as $F_{Treprostinil(30min)} / (F_{CytoD} - F_{initial}) \times 100$. C) Effects of DMSO load control on PASMC:HDF duo-culture, with doses added sequentially after 30 min incubation. **p<0.01, ****p<0.0001 as determined by one-way ANOVA. All plots mean ± S.E.M and each data point indicating an individual microtissue.

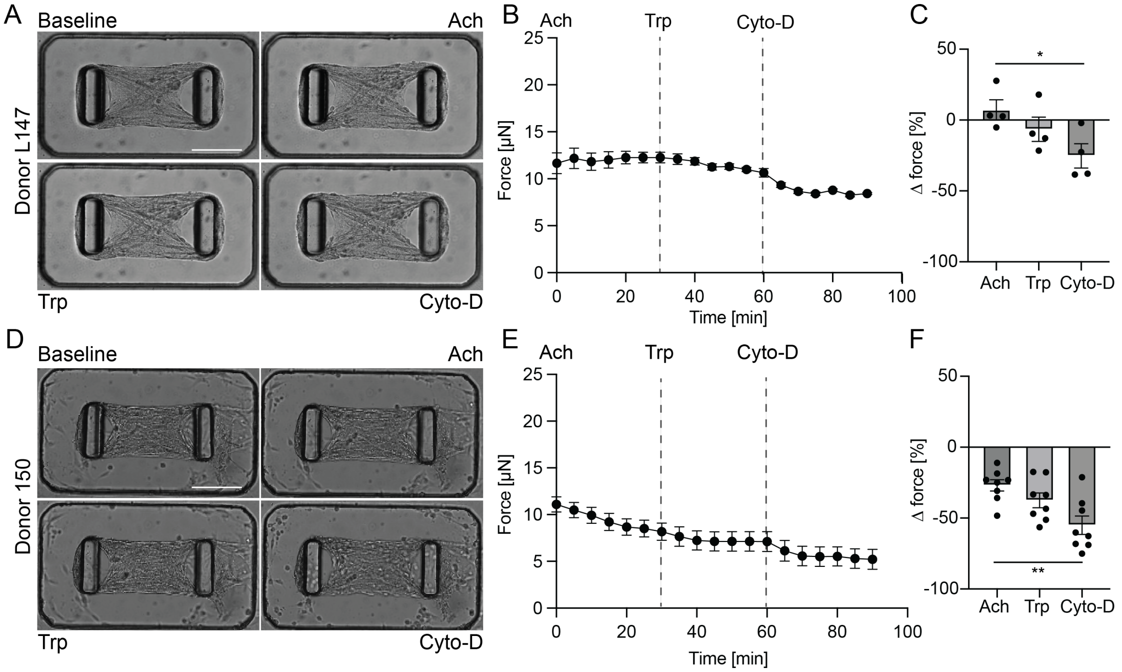

Figure S8. Dynamic drug treatment of donor PA-SMUGs. (A) Representative phase-contrast images of donor L147 PASMC:HDF (4:1) microtissues at baseline and after sequential acetylcholine (Ach), Treprostinil (Trp), and cytochalasin-D (Cyto-D) treatments. (B) Dynamic force measurements of donor L147 PASMC:HDF microtissues throughout drug treatments at timepoints indicated on graph. (C) Changes in contractile force of donor L147 PASMC:HDF microtissues after 30 min drug treatments normalized to baseline contraction values prior to drug treatment (negative values indicate microtissue relaxation). D) Representative phase-contrast images of donor 150 PAEC:HDF (4:1) microtissues at baseline and after drug treatments as in (B). (E) Dynamic force of donor 150 PAEC:HDF microtissues in response to drug treatment. (F) Changes in contractile force of donor 150 PAEC:HDF microtissues after 30 min drug treatments. Scale bar 0.24 mm. All plots are mean ± S.E.M. from n ≥ 4 microtissues, with individual datapoints referring to individual microtissues. *$p<0.05$, **$p<0.01$, as determined by one-way ANOVA.

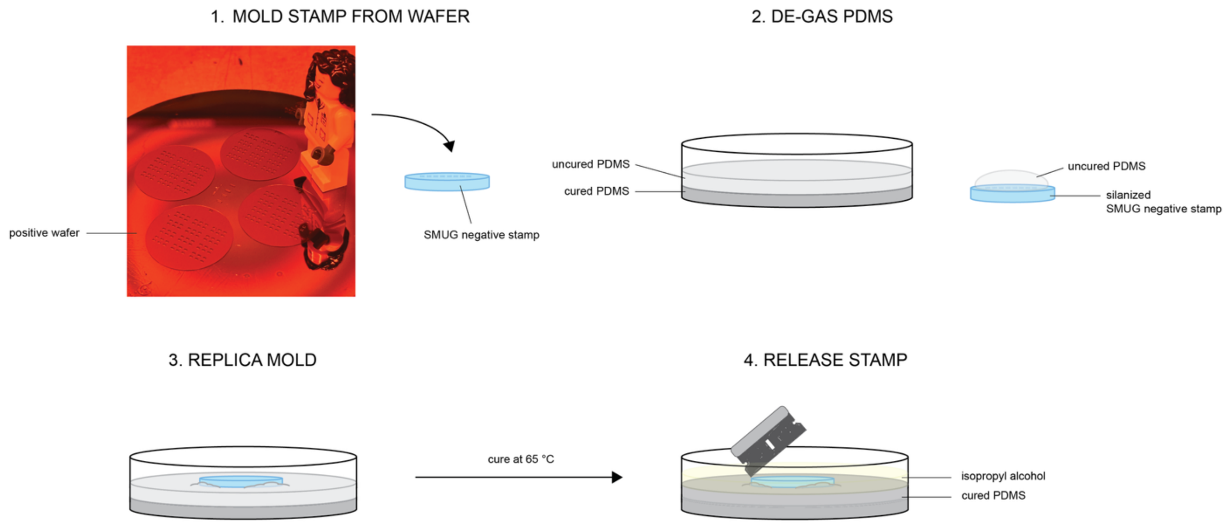

Figure S9. Replica molding method for generating positive PDMS structures. A layer of cured PDMS in a 15 mm dish prevents tissues from contacting untreated plastic. A SMUG negative stamp pretreated with Trichloro(1H, 1H, 2H, 2H-perfluorooctyl)silane is coated with PDMS and placed into a layer of uncured PDMS, creating a recessed well with positive SMUG microcantilevers.

Supplementary Video 1. Timelapse video of microtissues during assembly. Differential interference contrast microscopy images of single slices of image z-stacks imaged at 10x magnification on a laser scanning confocal microscope. Frames are every 15 minutes for a total of 18 hrs. Tissues are PA-SMUG triculture seeded with PAEC, PASMC, and HDF. Force measurements are provided in Fig. S5.

Supplementary Video 2. Timelapse video of cell migration during tissue assembly. 3D rendering of PA-SMUG triculture microtissues seeded with PAEC (tracks and cells shown in grey) and PASMC (tracks and cells shown in red) labeled with SpyFast cytoskeletal dyes and unlabeled HDF. Microtissue was imaged at 10x with a laser scanning confocal microscope, and frames are every 15 minutes for a total of 18 hrs.

2. **Supplementary Methods**

1.1 Gel contraction assay

BSMC- and PASMC-laden collagen type I hydrogels were added to low-attachment 96-well plates (Corning) at a cell concentration of 50,000 cells in 100 µL of 2.5 mg/mL collagen pre-gel slurry. The collagen slurry was polymerized at 37 °C for 20 min in a tissue culture incubator, then SmGm-2 was added to each well along with drug treatment where indicated, which was added to media once daily. Phase contrast images were acquired at 4x magnification and stitched in Fiji, with regions of interest to quantify visible hydrogel area. Discontinuous hydrogel fragments were not included in quantitation for treatments that elicited gel degradation.

1.2 Multi-channel microfluidic devices

Master molds for microfluidic devices were fabricated using photoresist-epoxy laminates (SU-EX, DJ Microlaminates, Sudbury, MA). Silicon wafers (100 mm, University Wafer), were first treated with 0.1% hydrofluoric acid etchant to strip the oxide layer then rinsed with isopropyl alcohol. A laminator heated to 65 °C with a rolling speed of 1 ft/min was used to laminate 150 µm SU-EX sheets on the silicon wafers. Laminated wafers were exposed through a transparency mask with collimated 365 nm light using a mask aligner (MA6/BA6, Karl Suss). A post-exposure bake at 65 °C for 30 min and 95 °C for 2 hrs was applied prior to development with SU-8 developer (Kayaku Advanced Materials). Developed wafers were passivated in vacuum deposition with trichloro-1H-1H-2H-2H-perfluorooctyl silane (Sigma-Aldrich, St. Louis, MO, USA). Microfluidic devices were fabricated from the master mold using standard soft lithography. Cell and collagen concentrations were the same as the gel contraction assay.

1.3 Fabrication parameters for SMUGs

Silicon master molds for stiff ($SMUG_{1.45}$) and compliant ($SMUG_{0.52}$) SMUGs were fabricated using standard photolithography and parameters described in Table S1.

Table S1. Fabrication parameters for SMUGs

Step	Procedure	Parameter	
		$SMUG_{1.45}$	$SMUG_{0.52}$
1.	Strip oxide layer	Clean wafer with dilute HF for 1 minute	
2.	Spin adhesion layer	Spin SU-8 2005 with a 2-step spin:<br>1. 500 rpm, 10 seconds, 84 rpm/s acceleration<br>2. 3000 rpm, 60 seconds, 252 rpm/s acceleration	
3.	Soft bake	2 minutes at 95 °C	
4.	Exposure	Flood expose:<br>104 mJ, no UV filter<br>Single-step exposure	
5.	Post-exposure bake	2 minutes at 95 °C	
6.	Spin pillar layer	Spin SU-8 2150 with a 2-step spin:<br>1. 500 rpm, 90 seconds, 84 rpm/s acceleration<br>2. 1100 rpm, 120 seconds, 336 rpm/s acceleration	
7.	Soft bake	10 minutes at 65 °C<br>30 minutes at 95 °C	

8.	Make blocking layer	70% SU-8 2010 (negative photoresist) + 30% S-1813 (positive photoresist)	50% SU-8 2010 (negative photoresist) + 50% S-1813 (positive photoresist)
9.	Spin blocking layer	Spin blocking layer with a 2-step spin:<br>1. 500 rpm, 120 seconds, 84 rpm/s acceleration<br>2. 1000 rpm, 30 seconds, 336 rpm/s acceleration	Spin blocking layer with a 2-step spin:<br>1. 500 rpm, 10 seconds, 84 rpm/s acceleration<br>2. 1000 rpm, 30 seconds, 336 rpm/s acceleration
10.	Soft bake	10 minutes at 65 °C<br>30 minutes at 95 °C	2 minutes at 65 °C<br>5 minutes at 90 °C
11.	Expose pillars	Expose through pillar mask:<br>324 mJ through UV-filter<br>Multiple exposure	Expose through pillar mask:<br>500 mJ through UV-filter<br>Multiple exposure
12.	Spin cap layer	Spin SU-8 2050 with a 2-step spin:<br>1. 500 rpm, 120 seconds, 84 rpm/s acceleration<br>2. 1250 rpm, 90 seconds, 252 rpm/s acceleration	
13.	Soft bake	10 minutes at 65 °C<br>30 minutes at 95 °C	
14.	Expose cap layer	Expose through cap mask:<br>400 mJ through UV-filter<br>Multiple exposure	Expose through cap mask:<br>175 mJ through UV-filter<br>Multiple exposure
15.	Post-exposure bake	10 minutes at 65 °C<br>30 minutes at 95 °C	
16.	Develop in PGMEA	15 minutes at 50 rpm on orbital shaker	30 minutes at 50 rpm on orbital shaker
17.	Rinse with IPA	2 minutes, or redeveloping until IPA runs clear	

1.4 Determination of SMUG cantilever stiffness
The stiffness of SMUG cantilevers was determined as previously described [Ref. 36] using the geometric parameters depicted in Fig. S2. To validate these measurements, a finite element model (FEM) of each pillar geometry was generated in COMSOL Microphysics (COMSOL Inc, Burlington, MA, USA). The model geometry was determined from confocal imaging of SMUG devices fabricated with Nile-red labeled PDMS (Fig. S2). The cantilever was modeled as a linear elastic solid with a density of 1000 kg/m^3, a Poisson's ratio of 0.45, and a Young's modulus of 1.61 MPa and was meshed using an extra-fine, physics-controlled mesh. To determine cantilever stiffness, a force of 20 μN was applied at the cantilever cap, and displacement was measured at the cap (Fig. S3). Using the model, we determined the stiffness of $SMUG_{0.52}$ to be 0.57 μN/μm and $SMUG_{1.45}$ to be 1.42 μN/μm, consistent with analytical calculations which resulted in stiffness values of 0.52 μN/μm and 1.42 μN/μm, respectively.

1.5 Donor cell information
Age and sex of IPAH donor cells as obtained from PHBI are indicated in Table S2.

Table S2. Cell source and donor characteristics for primary smooth muscle cells.

Abbreviation	Age	Sex	Disease	Source
PASMC Donor 82	25	Male	IPAH	PHBI
PASMC Donor L147	39	Female	IPAH	PHBI
PASMC Donor 11	58	Female	IPAH	PHBI
PASMC Donor 150	16	Female	IPAH	PHBI
PASMC Donor 13	15	Female	IPAH	PHBI

1.6 Live-cell imaging of SMUG assembly

To track cell dynamics during microtissue assembly, SMUG devices were fabricated and prepared as described in the main methods and seeded according to the ratios and protocols described in the SMUG seeding section of the main methods. Prior to seeding, PASMC were labeled with SPY555-actin (Cytoskeleton Inc., Denver, CO) for 1 hr and PAEC were labeled with SPY650-FastAct (Cytoskeleton Inc.) for 2 hr according to manufacturer specifications. After seeding, devices were transferred to a live-cell chamber (Tokai Hit, Bala Cynwyd, PA) maintained at 37 °C and 5 % CO_2 on a laser scanning confocal microscope (Olympus FV3000) with a programmable stage. Individual tissues were imaged with a 10x U Plan S-APO, 0.4 NA air objective every 15 min for 18 hrs. Individual cell tracks were determined using the Add Spots function in Imaris (Oxford Instruments, Morrisville, NC).
